# IL-6 Degradation by Secreted Proteases From *Paracoccidioides restrepiensis*

**DOI:** 10.1155/ijm/5566307

**Published:** 2025-10-09

**Authors:** Priscila de Oliveira, Bianca Carla Silva Campitelli Barros, Maria Aparecida Juliano, Aparecida Sadae Tanaka, Adriana Karaoglanovic Carmona, Saara Maria Batista dos Santos, Paloma Korehisa Maza, Rosana Puccia, Alexandre Keiji Tashima, Erika Suzuki

**Affiliations:** ^1^Department of Microbiology, Immunology, and Parasitology, Paulista School of Medicine, Federal University of São Paulo, São Paulo, Brazil; ^2^Department of Biophysics, Paulista School of Medicine, Federal University of São Paulo, São Paulo, Brazil; ^3^Department of Biochemistry, Paulista School of Medicine, Federal University of São Paulo, São Paulo, Brazil

**Keywords:** fungal proteases, IL-6 degradation, mass spectrometry, *Paracoccidioides restrepiensis*

## Abstract

Paracoccidioidomycosis is a systemic fungal disease caused by *Paracoccidioides* spp., predominantly affecting populations in Latin America, with Brazil reporting the highest number of cases. The infection is associated with severe pulmonary and systemic manifestations. Previous studies have highlighted the role of fungal proteases in adhesion, invasion, and the modulation of host immune responses, implicating them as key virulence factors. Our group previously demonstrated that *Paracoccidioides restrepiensis* secretes proteases that activate protease-activated receptors (PAR-1 and PAR-2) in human lung epithelial cells, stimulating the secretion of proinflammatory cytokines, including IL-6 and IL-8. We hypothesized that *P. restrepiensis* secretes proteases that are capable of degrading key host cytokines, such as IL-6, thereby contributing to modulate the host immune response during infection. This study is aimed at identifying and characterizing proteases secreted by *P. restrepiensis* that degrade human IL-6. Proteases secreted by *P. restrepiensis* were isolated using a *p*-aminomethylbenzamidine (*p*ABA)-Sepharose affinity column. Protease-containing fractions were incubated with recombinant human IL-6 and further analyzed by Western blot to evaluate their ability to degrade this cytokine. Fractions were submitted to liquid chromatography and mass spectrometry to characterize the proteome content, focusing on the identification of fungal proteases. The hydrolysis of IL-6 in the presence of different protease inhibitors was also analyzed to confirm the specific activity of the fungal proteases. Enzymatic assays revealed proteases that hydrolyze human IL-6, suggesting a mechanism by which *P. restrepiensis* modulates the host immune response. In addition, mass spectrometry analysis confirmed the presence of a serine protease in the protease activity–containing fractions. These findings indicate that *Paracoccidioides* proteases may modulate host immune response by degrading key cytokines involved in inflammation and host defense.

## 1. Introduction

Paracoccidioidomycosis (PCM) is a human systemic mycosis caused by the thermally dimorphic fungi of the *Paracoccidioides* genus. PCM is prevalent across Latin American countries, but Brazil accounts for the highest number of reported cases [1]. In Brazil, as PCM was not a notifiable disease until 2020, numbers might be underestimated, leading to an inaccurate epidemiology [2]. Currently, the genus *Paracoccidioides* comprises five species, which are classified based on their genetic variability: *P. lutzii*, *P. brasiliensis* sensu stricto, *P. americana*, *P. restrepiensis*, and *P. venezuelensis* (the latter four species belong to the *P. brasiliensis* complex) [3]. Nonetheless, there are no differences in PCM clinical manifestations caused by the different *Paracoccidioides* species [4].

The establishment, progression, and severity of an infection depend on factors associated with both the host's immune response and the pathogen's virulence. In this context, fungal pathogens have evolved by refining their pathogenic mechanisms to evade host defenses [5]. For example, proteases are produced by pathogenic fungi and participate in the modulation of essential infection processes, such as adhesion and invasion of the host tissues [5]. Serine proteases of *Cryptococcus neoformans*, for instance, degrade structural epithelial components, thus facilitating the invasion and dissemination processes in the host [6]. Castilho and coworkers [5] demonstrated that mice immunization with a recombinant aspartyl protease from *P. brasiliensis* conferred a protective effect against experimental PCM. These animals showed a decrease in fungal burden as well as the absence of parenchyma injuries. In addition, the authors verified that Pepstatin A, an aspartyl protease inhibitor, decreased injury and fungal loads in mice lungs, suggesting that *Paracoccidioides* aspartyl protease may be involved in the virulence of this fungus [5].

It has also been demonstrated by several groups that fungi secrete proteases that may activate host immune cells, leading to the release of cytokines and chemokines [7]. Over the past decade, our group has reported that different *Paracoccidioides* species induce secretion of interleukin (IL)-6 and IL-8 by the human lung epithelial cell line A549 [8–12]. We also observed that *P. restrepiensis*, for example, secretes serine and cysteine proteases that induce IL-6 and IL-8 secretion by A549 cells in a Protease-Activated Receptor (PAR)-1 activation– and PAR-2 activation–dependent manner [11].

Besides leading to cytokine secretion, proteases from several pathogens may also modulate host immune responses by inactivating, for example, components of the complement system, immunoglobulins, and cytokines [13–15]. Proteases that cleave several cytokines have been described for bacteria [16]. Theander et al. have shown that elastase and alkaline protease of *Pseudomonas aeruginosa* inhibited the proliferation of murine lymphocytes [17]. This inhibition was promoted by the cleavage of IL-2, since the authors detected degradation products of this cytokine and found that preincubation of IL-2 with proteases reduced cytokine binding to its receptor [17]. While *Porphyromonas gingivalis* expresses cysteine proteases, called gingipains, which cleave IL-6, IL-8, and TNF-*α* [18], some of the *Legionella pneumophila* proteases degrade IL-2 and TNF-*α* [16, 19]. Regarding fungi, our group verified that when incubating fixed *P. brasiliensis* yeasts with A549 epithelial cells, the culture supernatant of these cells presented higher concentrations of IL-6 and IL-8 when compared to the supernatant of the cells incubated with live yeasts of this fungus [8]. Indeed, we found that yeast proteases, expressed by *P. brasiliensis*, promoted degradation of these cytokines. However, it was not possible to determine whether this protease was secreted into the culture supernatant or whether it was expressed on the fungus surface [8].

In the present study, we report that *P. restrepiensis* yeasts (Pb339) secrete proteases that promote IL-6 degradation. By using a *p*-aminomethylbenzamidine (*p*ABA)-Sepharose column, we isolated fractions containing protease activity (PrP) from *P. restrepiensis* culture supernatant. Moreover, by ultraperformance liquid chromatography coupled to a mass spectrometer (UPLC/HDMS), we identified peptide products obtained from hrIL-6 hydrolysis and a *Paracoccidioides* serine protease that potentially may be involved in IL-6 degradation.

## 2. Materials and Methods

### 2.1. Fungal Growth Conditions

*P. restrepiensis*, isolate Pb339, was kindly provided by Dr. Zoilo P. Camargo, Universidade Federal de São Paulo, São Paulo, Brazil. Yeasts were grown as described previously [11]. Briefly, fungal aliquots were cultivated in modified YPD medium (0.5% (*w*/*v*) peptone, 1.5% (*w*/*v*) glucose, and 0.5% (*w*/*v*) yeast extract) for 5–7 days in a shaker incubator at 37°C, 100 rpm. Under these conditions, the culture is in the late log phase, and the components found in the supernatant are not the product of cell death [20]. Fungal culture was processed in the log phase of growth, avoiding the presence of cell lysis products in the supernatant. Peptone and yeast extract were purchased from BD Biosciences (United States), and glucose was purchased from Sigma-Aldrich (United States).

### 2.2. Isolation of *P. restrepiensis* Proteases

*P. restrepiensis* yeasts were cultured for 7 days and filtered (pore size 0.4 *μ*m; Corning, United States) to obtain cell-free culture supernatant. Twenty-five milliliters of this culture supernatant were buffered with 25 mL of 50 mM Tris–HCl, pH 8.0, containing 1 M NaCl, and loaded into an affinity column (1 mL) of *p*ABA-Sepharose (Pharmacia LKB, Sweden). After a washing step with 10 volumes of the same buffer, bound compounds were eluted with 50 mM glycine, pH 3.0. Ten fractions of 1 mL were collected into tubes containing 200 *μ*L of 1 M Tris. To detect proteolytic activity in the eluted fractions, aliquots were incubated at 37°C for 4 h with 100 ng of hrIL-6, and the degradation of this cytokine was evaluated by Western blot (as described below), using horseradish peroxidase–conjugated anti-IL-6 antibodies. The fraction that showed the highest proteolytic activity over IL-6 degradation was named *P. restrepiensis* protease–containing fraction (PrP).

### 2.3. Analysis of the Proteolytic Degradation of Human Recombinant (hr)IL-6 by *P. restrepiensis* Proteases

To obtain *P. restrepiensis* culture supernatant, yeasts were cultured for 7 days and filtered (pore size 0.4 *μ*m; Corning, United States). To evaluate hrIL-6 degradation by *P. restrepiensis* secreted proteases, 14 *μ*L of culture supernatant were incubated at 37°C for 4 h with 100 ng of hrIL-6. For experiments with protease inhibitors, aliquots of *P. restrepiensis* culture supernatant or *P. restrepiensis* Protease Activity–Containing Fraction 4 (F4) (PrP) were incubated for 20 min, at 37°C with (i) saline solution (NaCl 0.9%) as vehicle for aprotinin (AP), (ii) 2 *μ*g/mL AP (serine protease inhibitor) in saline solution, (iii) 6.6% ethanol in water as vehicle for Pepstatin A, (iv) 2 *μ*g/mL Pepstatin A in 6.6% ethanol solution, (v) TN (Tris–HCl 300 mM and NaCl 50 mM, pH 8) solution as vehicle for BmSI (subtilisin protease inhibitor [21]), (vi) 200 nM BmSI in TN solution, (vii) 24 *μ*M *p*-HMB (*p*-hydroxymercuribenzoic acid—a serine and cysteine protease inhibitor), (viii) 40 *μ*M leupeptin (Leu) (a serine and cysteine protease inhibitor), (ix) 40 *μ*M AEBSF (4-[(2-aminoethyl]benzenesulfonyl-fluoride-hydrochloride—a serine protease inhibitor), (x) 400 *μ*M EDTA (ethylenediamine tetraacetic acid—a metalloprotease inhibitor), or (xi) 3 *μ*M E-64 (*trans*-epoxysuccinyl-L-leucylamido-[4-guanidino] butane—a cysteine protease inhibitor). After 20 min, 100 ng of hrIL-6 were added to each sample, incubated for 4 h at 37°C, and the IL-6 degradation was evaluated by SDS–Tricine PAGE [22], followed by Western blot using anti-IL-6 antibodies (Invitrogen, EUA), as described previously [23]. Reactive proteins were detected using a chemiluminescent reagent (SuperSignal West Pico Chemiluminescent Substrate, Pierce, United States) and documented by UVITEC (United Kingdom).

### 2.4. Mass Spectrometry Analysis

Aliquots of 500 ng of hrIL-6 were incubated at 37°C with 40 *μ*L of PrP for 4 h. Samples (three technical replicates) were analyzed in a Synapt G2 HDMS Q-TOF mass spectrometer (Waters) coupled to a nanoAcquity UPLC chromatographic system. Samples were injected into a trap column (nanoAcquity C18 trap column Symmetry 180 *μ*m × 20 mm, Waters) and transferred by an elution gradient to an analytical column (nanoAcquity C18 BEH 75 *μ*m × 150 mm, 1.7 mm, Waters). Buffers A (0.1% formic acid in water) and B (0.1% formic acid in acetonitrile) were used to generate a 7%–35% B elution gradient run over 92 min at a flow rate of 275 nL/min. Data were acquired by HDMS mode, switching from low (4 eV) to high (ramped from 19 to 45 eV) collision energy for accurate measurement of both intact peptides and fragments. Glu-Fibrinopeptide B (Waters) was infused using a nanoLockSpray apparatus and scanned every 30 s for external calibration.

ProteinLynx Global Server Software Version 3.0.1 (Waters) was used for mass spectrometry data processing and for database search against human cytokine sequences in the UniProtKB/Swiss-Prot database (http://www.uniprot.org, including 1659 entries) in PEAKS Studio 7.5 software. The following search parameters were used: carbamidomethylation of cysteine as a fixed modification (+57.02 kDa) and oxidation of methionine (+15.99 kDa), N-terminal acetylation (+42.01 kDa), glutamine and asparagine deamidation (+0.98 kDa) as variable modifications (PTMs), up to 2 missed cleavage sites were allowed for trypsin digestion and automatic fragment and peptide mass tolerance. The following criteria were set for protein identification: a minimum of one fragment ion per peptide, five fragment ions per protein, and two peptides per protein, and the false discovery identification rate (FDR) was set to 1%, estimated by simultaneous search against a reversed database (decoy). The molecular weights of the identified peptides were determined using the Expasy tool, available at http://web.expasy.org/peptide_mass.

### 2.5. In-Solution Protein Digestion

Fifty microliters of PrP were diluted in 50 mM NH_4_HCO_3_ for digestion with trypsin (enzyme-to-substrate ratio 1:100), as previously described [24]. Briefly, all samples were reduced with 5 mM dithiothreitol for 30 min at 60°C and alkylated with 14 mM iodoacetamide for 30 min in the dark at room temperature. Incubations with trypsin were conducted at 37°C overnight. The reactions were stopped with 0.5% trifluoroacetic acid. Samples were filtered through 0.22-*μ*m syringe filters and placed in glass vials for further LC-MS/MS analysis.

### 2.6. LC-MS/MS Analysis

The LC-MS/MS analyses of peptides from in-solution digested proteins were performed on a Synapt G2 HDMS mass spectrometer coupled to a nanoAcquity liquid chromatography system (Waters, United States). Tryptic peptides were loaded and desalted for 5 min in a Symmetry C18 trap column (5 *μ*m particles, 180 *μ*m × 20 mm, Waters) at a flow rate of 8 *μ*L/min of Phase A (0.1% formic acid). Then, they were subsequently separated by elution with a gradient of 7%–35% of Phase B (0.1% formic acid in acetonitrile) through a C18 nanoAcquity BEH 130 capillary column (1.7 *μ*m particles, 75 *μ*m × 150 mm, Waters) in 60 min at a flow rate of 275 nL/min. Data were acquired in the data-independent mode UDMS^E^ [25], with a *m*/*z* range of 50–2000 and in the resolution mode. Collision energies were alternated between 4 eV and a ramp of 17–60 eV for precursor ion and fragment ions, respectively, using scan times of 1.25 s. The ESI source was operated in positive mode with a capillary voltage of 3.0 kV, block temperature of 100°C, and cone voltage of 40 V. For lock mass correction, [Glu1]-Fibrinopeptide B solution (500 fmol/mL in 50% acetonitrile, 0.1 formic acid; Waters, Milford, Mssachusetts, United States) was infused through the reference sprayer at 500 nL/min and sampled every 60 s. Samples were analyzed in duplicates.

### 2.7. Data Processing and Protein Identification

The mass spectrometer raw data were processed in ProteinLynx Global Server 3.0.3 (Waters) using a low energy threshold of 750 counts and an elevated energy threshold of 50 counts. The MS/MS spectra were exported as mzML files and imported into PEAKS Studio 7.5 (Bioinformatics Solution Inc., Waterloo, Canada) [26] for de novo analysis. The *Paracoccidioides brasiliensis* (strain Pb18) reviewed database (8399 entries, downloaded on February 13, 2017, from https://www.uniprot.org) was used as a reference bank. De novo analysis and database search were performed with precursor mass tolerances of 10 ppm and fragment mass tolerance of 0.025 Da. Carbamidomethylation of cysteine (+57.02 kDa) was set as a fixed modification, and oxidation of methionine (+15.99 kDa) was set as a variable modification. A maximum of two missed cleavages for trypsin were accepted. The peptide FDR was estimated by the decoy fusion method [26] and was set at a maximum of 1%.

## 3. Results

### 3.1. IL-6 Degradation by Components of the *P. restrepiensis* Culture Supernatant

**Figure 1:**
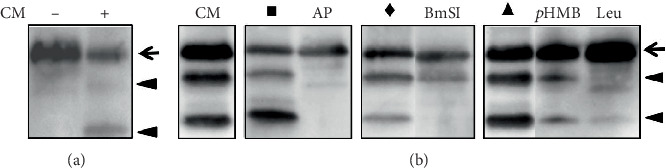
Western blot analysis of hrIL-6 degradation after incubation with *P. restrepiensis* culture supernatant. (a) Human recombinant (hr) IL-6 was incubated in the absence (−) or presence (+) of *P. restrepiensis* culture supernatant (CM). (b) hrIL-6 was incubated with *P. restrepiensis* culture supernatant (CM) preincubated with the serine protease inhibitor aprotinin (AP), the subtilisin-like serine protease inhibitor from *Boophilus microplus* (BmSI), the cysteine protease inhibitor *p-*(hydroxymercuri)benzoic acid (*p*-HMB), or the cysteine–serine–threonine protease inhibitor leupeptin (Leu). Controls were performed without inhibitor vehicle (CM) or with (∎) ultrapure water (used as a vehicle for AP); (♦) 50 mM NaCl with Tris–HCl 300 mM, pH 8.0 (used as a vehicle for BmSI); and (▲) benzyl alcohol (used as a vehicle for *p*-HMB and Leu). In (a) and (b), the hrIL-6 (21.3 kDa, arrow) and hrIL-6 cleavage products (arrowheads) were detected by anti-hrIL-6 antibodies. This result is representative of three independent experiments.

Aliquots of the *P. restrepiensis* culture supernatants were incubated with human recombinant (hr)IL-6, and the degradation profile of this cytokine was analyzed by SDS–Tricine PAGE followed by Western blot. Under our experimental conditions, we observed a reduction in the intensity of the hrIL-6 band (21.3 kDa, arrow) and the generation of lower molecular mass products (arrowheads) (Figure 1a), indicating that the *P. restrepiensis* yeasts secrete components, possibly proteases, that lead to hrIL-6 degradation.

To evaluate the type of proteolytic activity involved, hrIL-6 was incubated with aliquots of *P. restrepiensis* culture supernatants pretreated with different protease inhibitors.  Figure 1b shows that the serine protease inhibitor AP inhibited the hrIL-6 degradation, considering that the lower molecular mass bands seen in the control (CM [conditioned medium]) are absent in this sample. The subtilisin-like serine protease inhibitor BmSI, the cysteine protease inhibitor *p*-HMB, and the serine–cysteine–threonine protease inhibitor Leu, on the other hand, partially inhibited the hrIL-6 degradation, since lower molecular mass products were still observed, though in lower amounts compared to their controls. Together, these results suggest that *P. restrepiensis* yeasts secrete serine and cysteine proteases that can cleave IL-6.

### 3.2. IL-6 Degradation by Enriched Fractions Containing *P. restrepiensis* Secreted Proteases

**Figure 2:**
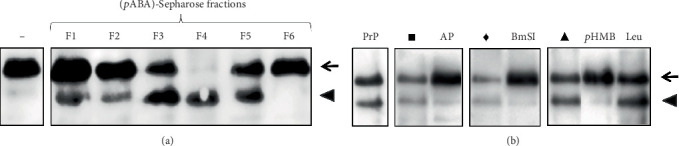
hrIL-6 degradation after incubation with (*p*ABA)-Sepharose eluted fractions. (a) hrIL-6 was incubated with elution buffer (−) or with (*p*ABA)-Sepharose eluted fractions (F1–F6). (b) hrIL-6 was incubated in the presence of Fraction 4, named PrP, pretreated with aprotinin (AP), the subtilisin-like serine protease inhibitor from *Boophilus microplus* (BmSI), *p-*(hydroxymercuri)benzoic acid (*p*-HMB), or leupeptin (Leu). Controls were performed without inhibitor vehicle (PrP) or with (∎) ultrapure water (used as a vehicle for AP); (♦) 50 mM NaCl in Tris–HCl 300 mM, pH 8.0 (used as a vehicle for BmSI); and (▲) benzyl alcohol (used as a vehicle for *p*-HMB and Leu). In (a) and (b), the cleavage products were detected by anti-hrIL-6 in a Western blot. Arrows show intact hrIL-6 (21.3 kDa), and arrowheads show hrIL-6 cleavage products.

As our results indicated that *P. restrepiensis* yeasts secrete serine proteases that promote hrIL-6 degradation, we used a *p*ABA-Sepharose column to enrich the proteolytic activity contained in the fungal culture supernatants. The eluted fractions were followed for proteolytic activity determination toward the cytokine hrIL-6, which was analyzed by Western blot. As shown in Figure 2a, the proteolytic activity was concentrated in F4, but it was also partially eluted in Fractions 1, 2, 3, and 5. Note that the intact hrIL-6 disappeared upon incubation with F4, while a lower molecular mass product was observed (Figure 2a, arrowhead). For this reason, F4 from the *p*ABA-Sepharose column isolation was named as the *P. restrepiensis* protease–containing fraction (PrP).

Preincubation of PrP with protease inhibitors showed that the degradation of hrIL-6 was inhibited by AP, BmSI, and *p-*HMB (Figure 2b). On the other hand, the presence of Leu did not prevent the degradation of the cytokine by PrP. Together, these results suggest that *P. restrepiensis* secretes serine and cysteine proteases that promote hrIL-6 degradation and can be enriched by chromatography in *p*ABA-Sepharose.

### 3.3. Analysis of hrIL-6 Cleavage Sites for PrP

**Figure 3:**
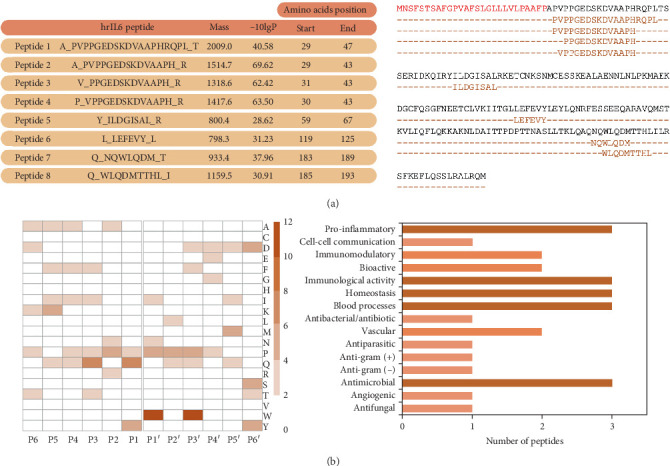
Analysis of the peptides obtained after incubation of hrIL-6 with *P. restrepiensis* protease–containing fraction (PrP). (a) Aliquots of PrP were incubated with hrIL-6 at 37°C for 4 h. Next, products were injected into a Waters nanoAcquity UPLC system coupled to a Synapt G2 HDMS. The data acquired by the mass spectrometer were processed, and searches were performed with the human IL-6 sequence in the UniProt database. Amino acids corresponding to the signal peptide in the IL-6 sequence are shown in red. Peptides identified by the mass spectrometer are shown in brown and aligned with the amino acid sequence of human IL-6 (in black). (b) hrIL-6 cleavage sites promoted by PrP. The heat map was produced using the CLIP-PICS web application and R 4.2.2. The prediction of peptide activity was performed using ProInflam (http://metagenomics.iiserb.ac.in/proinflam/index.html) and Peptipedia (https://peptipedia.cl/). Similar results were obtained in two other independent experiments.

The cleavage products obtained after the incubation of hrIL-6 with PrP were analyzed by UPLC/HDMS. Data obtained by the mass spectrometer were processed by ProteinLynx GlobalServer, and searches were performed comparing the sequences of 212 amino acids from human IL-6 in the UniProt database (UniProt ID P05231, accessed in October 2022). The data processed by the software, the sequence and mass of the peptides, experimental error (−10 lgP), and position of the amino acids in the respective IL-6 sequence are shown in Figure 3a. We were able to identify the presence of eight peptides after the hydrolysis of hrIL-6 by PrP. This result indicates that PrP contains proteases that cleave hrIL-6 at several sites, generating peptides with different molecular weights.

To analyze the relative occurrence of each amino acid residue in P6-P6', a heat map was generated with the peptides resulting from hrIL-6 hydrolysis by PrP (Figure 3b). A preference for tryptophan at P1' and P3' was observed, indicating peculiarities regarding the activity of PrP proteases involved in hrIL-6 hydrolysis. In general, these results indicate that PrP promotes IL-6 degradation with no clear preference for a specific amino acid as a substrate, suggesting the involvement of at least two different protease activities.

To predict the biological activity, including the proinflammatory properties of the peptides generated after hrIL-6 cleavage by PrP, the sequences were submitted to the MetaBioSys ProInflam tool (available at http://metagenomics.iiserb.ac.in/proinflam/index.html) and to the Peptipedia tool (available at https://peptipedia.cl), considering a confidence threshold value ≥0.87. Of the eight peptides identified, the sequences of Peptides 2, 4, and 6 showed a predicted proinflammatory function. Other biological activities predicted include homeostasis, blood processes, antimicrobial, and immunological activities (Figure 3b).

### 3.4. Identification of Proteases by LC-MS/MS From *P. restrepiensis* Protease–Containing Fraction (PrP)

**Figure 4:**
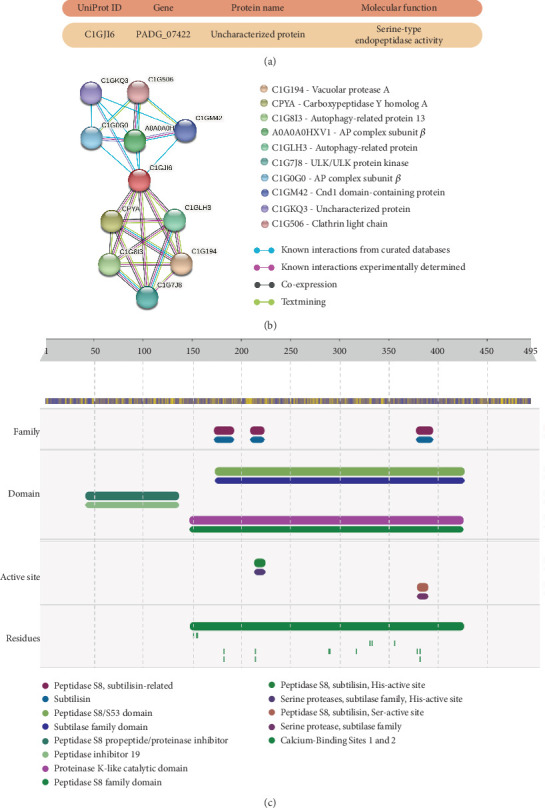
Characteristics of the protease identified by LC-MS/MS in *P. restrepiensis* protease–containing fraction (PrP). PrP was injected into a Waters nanoAcquity UPLC system coupled with a Synapt G2 HDMS. The data acquired by the mass spectrometer were processed, and then, searches were performed with *Paracoccidioides brasiliensis* isolate 18 in the UniProt database. (a) Identified protein and its respective molecular function, which was named *P. brasiliensis* subtilisin–like serine protease (PbSSP). (b) PbSSP (C1GJI6) interaction map predicted by the String 11.5 tool (available at https://string-db.org). (c) Representation of families, domains, active sites, and residues observed at the PbSSP protein sequence by the InterPro tool (https://www.ebi.ac.uk/interpro/).

Aiming to identify the composition of PrP, aliquots of PrP were subjected to LC-MS/MS, the data were processed by ProteinLynx GlobalServer, and searches were performed with the sequence of *P. brasiliensis* isolate 18 (as a reference sequence) proteases in the UniProt database. In this analysis, we identified the presence of an uncharacterized protein with a peptide signal and UniProt ID C1GJI6 (Figure 4). After the identification, the protein was searched at String Version 11.5 and InterPro databases to verify the potential partners of interaction and signaling, as well as the classification of the protein sequence by predicting the presence of domains and functional sites (Figure 4).

As shown in Figure 4b, it is predicted that the identified protein interacts with proteins involved in autophagy (C1G8I3, C1GLH3, and C1G7J8), vesicle-mediated transport (A0A0A0HXV1, C1G0G0, C1GM42, and C1G506), and other proteases from different families (C1G194, CPYA, and C1GKQ3).  Figure 4c shows domains, active site, and residues present in the protein identified in PrP by LC-MS/MS. According to the InterPro tool, this protein is a protease with subtilase and peptidase S8/S53 family domains (sequence position 174-427), as well as their respective active sites (sequence position 214-224), which classify it as a serine protease subtilisin-like protein. Thus, we named the identified uncharacterized protein as *P. brasiliensis* subtilisin–like serine protease (PbSSP).

### 3.5. Comparative Analysis of PbSSP With Other Fungal Proteases Containing the Subtilisin Domain

A BLAST search (available at https://blast.ncbi.nlm.nih.gov/Blast.cgi) of the PbSSP (UniProt ID C1GJI6) showed a high score identity with other serine proteases from fungal species. This protein has shown 85% identity with the amino acid sequence from Aqualysin 1 from *Paracoccidioides lutzii* (UniProt ID C1H074), 71% identity with cerevisin from *Blastomyces dermatitidis* (UniProt ID F2TE84), and 70% identity with the Hc serine protease sequence from *Histoplasma capsulatum* (UniProt ID F0ULK1), all of them described as secreted subtilisin-like serine proteases. The alignment of amino acid sequences in different fungal species was obtained by using the Clustal O (1.2.4) multiple sequence alignment tool, and it is shown in Figure S1.

**Figure 5:**
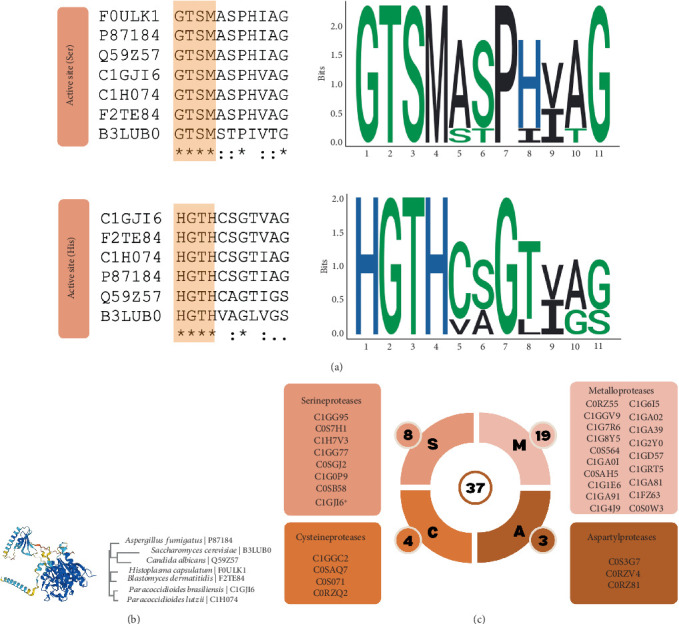
Comparative alignment of the PrP subtilase domain of PbSSP (UniProt ID C1GJI6) with others from well-characterized subtilisin-like serine proteases from fungi of clinical importance. (a) Serine (Ser) and histidine (His) active sites from serine proteases from *Paracoccidioides brasiliensis* (UniProt ID C1GJI6), *Paracoccidioides lutzii* (UniProt ID C1H074), *Blastomyces dermatitidis* (UniProt ID F2TE84), *Histoplasma capsulatum* (UniProt ID F0ULK1), *Aspergillus fumigatus* (UniProt ID P87184), *Candida albicans* (UniProt ID Q59Z57), and *Saccharomyces cerevisiae* (UniProt ID B3LUB0) were obtained at InterPro (https://www.ebi.ac.uk/interpro/). Multiple sequence alignment was performed at CLUSTALW (https://www.genome.jp/tools-bin/clustalw). ⁣^∗^, residues identical in all sequences tested, conserved substitutions. •, semiconserved substitutions. SeqLogo was constructed using the R language Version 4.2.2 and the package seqlogo. Basic amino acids are shown in blue, hydrophobic amino acids are shown in black, and polar amino acids are shown in green. (b) Analysis of the phylogenetic distribution of subtilase family domains from different fungi was performed using the Simple Phylogeny tool (available at http://www.ebi.ac.uk/Tools/phylogeny/simple_phylogeny/). (c) Schematic representation of characterized proteases from the genus *Paracoccidioides* available at MEROPS 12.4 (available at https://www.ebi.ac.uk/merops/index.shtml). The asterisk represents PbSSP (UniProt ID C1GJI6) in PrP, which is not currently included in the database.

In order to verify similarities among subtilase active sites at serine and histidine residues of PbSSP and different fungal species, sequences obtained at InterPro (available at https://www.ebi.ac.uk/interpro/) were submitted to Clustal W (available at https://www.genome.jp/tools-bin/clustalw), and the Logo comparative sequence alignment is shown in Figure 5a. Sequence conservation was observed among serine and histidine active sites of all proteins analyzed and comprised the residues GTSM for the serine active site and HGTH for the histidine active site. Conserved residues at active sites are used by proteases as specific motifs, playing a central role in the cleavage of peptide bonds [27].

By analyzing the phylogenetic distribution of subtilase family domains from different fungal species (Figure 5b), it was possible to verify that the sequence of the subtilase family domain from *P. brasiliensis* is very similar to that of *P. lutzii*, and sequences from this genus are closer to *Blastomyces dermatitidis* and *Histoplasma capsulatum*, two other dimorphic fungi, when compared to the subtilase domain sequences from *Candida albicans*, *Saccharomyces cerevisiae*, and *Aspergillus fumigatus* (Figure 5b). These findings are in consonance with the distribution of these microorganisms according to their taxonomy [28].

According to the MEROPS database, of the 37 sequences coding for known or putative proteases for the genus *Paracoccidioides*, 19 are classified as metalloproteases, 4 as cysteine proteases, 3 as aspartyl proteases, and 7 as serine proteases. Since comparable characteristics of PbSSP (UniProt ID C1GJI6) and other serine proteases are observed in the present work, we included this protein as a serine protease from *Paracoccidioides* (Figure 5c).

## 4. Discussion

Proteases secreted by pathogens have been identified as important virulence factors, being able to participate directly in the host immune response and playing a vital role in fungal pathogenesis by facilitating invasion, colonization, and dissemination of the fungus within the host. Among the mechanisms, the degradation of components that may modulate the immune system, such as receptors for immunoglobulins, receptors for cytokines, proteins of the complement system, proteins involved in the coagulation cascade, chemokines, or cytokines, is already described [13–16].

In our previous work, we reported that proteases of *Paracoccidioides* yeasts can also induce IL-6 and IL-8 release by A549 cells [11]. However, concomitantly, we observed that the fungus promoted the degradation of these cytokines [8]. The results of Maza et al. at that time did not clarify if the proteases that degraded IL-6 and IL-8 were present on the surface of the fungus or secreted [8]. We presently showed that *P. brasiliensis* yeasts secrete proteases that degrade IL-6. Behnsen and coworkers have shown that Alp1, a subtilisin/peptidase of the S8 family serine proteases, produced by *Aspergillus fumigatus*, can degrade host immune molecules such as immunoglobulins and complement proteins [14], which can impair the host immune response, allowing the fungus to evade detection and establish a persistent infection.

Concerning the degradation of cytokines, it has already been observed that some bacteria express proteases that degrade cytokines and, in this way, participate directly in pathogenesis. Okuda et al., for example, demonstrated that *P. aeruginosa* expresses the serine protease MucD that degrades IL-8, which in turn is important for the neutrophil transmigration to the site of infection [29]. Regarding *Porphyromonas gingivalis*, it has been seen that this bacterium increases the expression of IL-8 during infection in oral epithelial cells. However, the authors also demonstrate that this bacterium expresses proteases that degrade IL-8 [30]. Indeed, Banbula and coworkers demonstrated that *P. gingivalis* secretes cysteine proteases that promote IL-6 degradation in different sites of the protein [31].

In the present work, we show that *P. restrepiensis* isolate Pb339 from the *P. brasiliensis* complex secretes to the culture supernatant proteases that hydrolyze IL-6. In addition, assays performed with protease inhibitors have indicated that this fungus secretes serine proteases that promote IL-6 degradation. We managed to isolate a *P. restrepiensis* culture supernatant fraction containing protease activities (PrP) that were able to hydrolyze IL-6. Our UPLC/HDMS analysis of the IL-6 cleavage sites promoted by PrP identified the formation of at least two peptides with a molecular mass of approximately 2 kDa. Based on this result, if we subtract the mass values of the identified peptides from the intact human recombinant IL-6 (21.3 kDa) and compare it with our results observed by Western blot, we can infer that the band of approximately 18 kDa observed in Western blot corresponds to the fragment of IL-6 hydrolyzed by PrP.

Although 53 ORFs (open reading frames) coding proteases were identified in the *P. brasiliensis* transcriptome, only a few proteases of this fungus were characterized [32]. The MEROPS database has shown 37 sequences related to known or putative proteases of *Paracoccidioides*, suggesting that seven of them code for serine proteases. By comparing the sequence of the identified PbSSP with other serine proteases from different fungal species, we observed a high score identity (up to 70%) with serine proteases from *P. lutzii*, *B. dermatitidis*, and *H. capsulatum*. The sequence alignment of their serine and histidine active sites has shown conservation in the residues GTSM and HGTH, respectively.

A fingerprint of the peptides resulting from hrIL-6 hydrolysis has shown a preference for tryptophan at P1' and P3', which indicates peculiarities regarding the activity of PrP proteases involved in hrIL-6 hydrolysis. This observation is in consonance with the literature, which describes that subtilisins do not report a well-established preference for a sequence pattern of cleavage [33]. Thus, the understanding behind the mechanisms involved in the substrate selectivity of subtilisin/peptidase S8 family serine proteases, including the PbSSP identified in PrP, needs further investigation.

Parente and coworkers identified a serine protease secreted by *P. brasiliensis*, which could be involved in nitrogen acquisition by the fungus. Moreover, this protease could interact with FKBP-peptidyl prolyl *cis*–*trans* isomerases and with cytoskeleton proteins, thus playing different roles in a range of cellular processes [34]. More recently, Pigosso and coworkers showed by immunohistochemical analysis of infected mice lungs that a serine protease is secreted by *P. brasiliensis* yeasts during in vivo infection [35]; however, to date, no proteolytic activity has been attributed to this serine protease.

In 1995, Carmona and colleagues characterized a secreted subtilisin-like thiol-dependent serine protease from yeast cells of the same isolate used in the present work, specifically, *P. brasiliensis* (now *P. restripiensis*) B-339 [36]. The characterization was performed using fluorescence resonance energy transfer (FRET) peptides flanked by *ortho*-aminobenzoic acid (Abz) and ethylenediamine dinitrophenyl (EDDnp). The enzyme hydrolyzed the peptide Abz-MKRLTL-EDDnp specifically at the L–T bond, at an optimum alkaline pH, and was irreversibly inhibited by PMSF, mercury acetate, and *p*-HMB. The serine–thiol protease activity was considered a virulence factor due to its capacity to selectively cleave molecules of the basal membrane, specifically, laminin, fibronectin, Type IV collagen, and proteoglycans [20]. The thiol-dependent serine protease activity could be stabilized by fungal neutral polysaccharides [37]; however, full purification has never been achieved due to loss of enzymatic activity following a sequence of chromatographic steps. The best result was obtained upon enrichment in a *p*ABA-Sepharose column [38], where strong proteolytic activity against Abz-MKRLTL-EDDnp and fibronectin was acid-eluted in a specific fraction. At the time, an LC-MS/MS analysis of this fraction identified three main proteins, with only two peptides matching subtilisins. In the present study, LC-MS/MS analysis of PrP aliquots identified a single protein, designated as PbSSP (UniProt ID: C1GJI6), which is a secreted subtilisin-like serine protease. It will be very interesting to find out if PbSSP and the previously characterized serine–thiol protease [36] correspond to the same *Paracoccidioides* subtilisin.

In summary, the present study has shown that secreted proteases from *P. restrepiensis* cleave IL-6 in different sites, generating peptides with potential biological activities, including anti-inflammatory properties. Among these secreted proteases, a subtilisin-like serine protease with high similarity to other serine proteases from different pathogenic fungi was detected, which was called PbSSP. From our knowledge, this is the first study that shows a fungal proteolytic activity on cytokines and, thus, *P. restrepiensis* proteases may participate in the modulation of the host immune response during infection, which highlights the complex interplay between fungal proteases and the host immune response.

## Data Availability

The data that support the findings of this study are available from the corresponding author upon reasonable request.

## Supporting Information

Supporting Information Additional supporting information can be found online in the Supporting Information section. Supporting data and raw files are available at ftp://massive-ftp.ucsd.edu/v09/MSV000097562/. Figure S1: Multiple sequence alignment of PbSSP from PrP with other characterized secreted serine proteases from dimorphic fungi. Similarity between serine protease sequences from distinct dimorphic fungi was obtained by using the Clustal O (1.2.4) multiple sequence alignment tool. PbSSP (UniProt ID C1GJI6) identified in PrP was used as the reference sequence, and it is shown at the first line. Sequences of serine proteases from *Paracoccidioides lutzii* (UniProt ID C1H074), *Blastomyces dermatitidis* (UniProt ID F2TE84), and *Histoplasma capsulatum* (UniProt ID F0ULK1) are shown in lines 2, 3, and 4, respectively. ⁣^∗^, residues identical in all sequences tested. :, conserved substitutions. •, semiconserved substitutions. ◆-◆ represents the subtilase domain for the reference sequence.
