# Supplementary material for: IL-6 Degradation by Secreted Proteases From Paracoccidioides restrepiensis
Source: Int J Microbiol. 2025 Oct 9;2025:5566307. doi: 10.1155/ijm/5566307 (PMC12530924; doi:10.1155/ijm/5566307)
Supplement: Supporting Information — Additional supporting information can be found online in the Supporting Information section. Supporting data and raw files are available at ftp://massive-ftp.ucsd.edu/v09/MSV000097562/. Figure S1: Multiple sequence alignment of PbSSP from PrP with other characterized secreted serine proteases from dimorphic fungi. Similarity between serine protease sequences from distinct dimorphic fungi was obtained by using the Clustal O (1.2.4) multiple sequence alignment tool. PbSSP (UniProt ID C1GJI6) identified in PrP was used as the reference sequence, and it is shown at the first line. Sequences of serine proteases from Paracoccidioides lutzii (UniProt ID C1H074), Blastomyces dermatitidis (UniProt ID F2TE84), and Histoplasma capsulatum (UniProt ID F0ULK1) are shown in lines 2, 3, and 4, respectively. ⁣∗, residues identical in all sequences tested. :, conserved substitutions. •, semiconserved substitutions. ◆-◆ represents the subtilase domain for the reference sequence. [file 5566307.f1.docx]

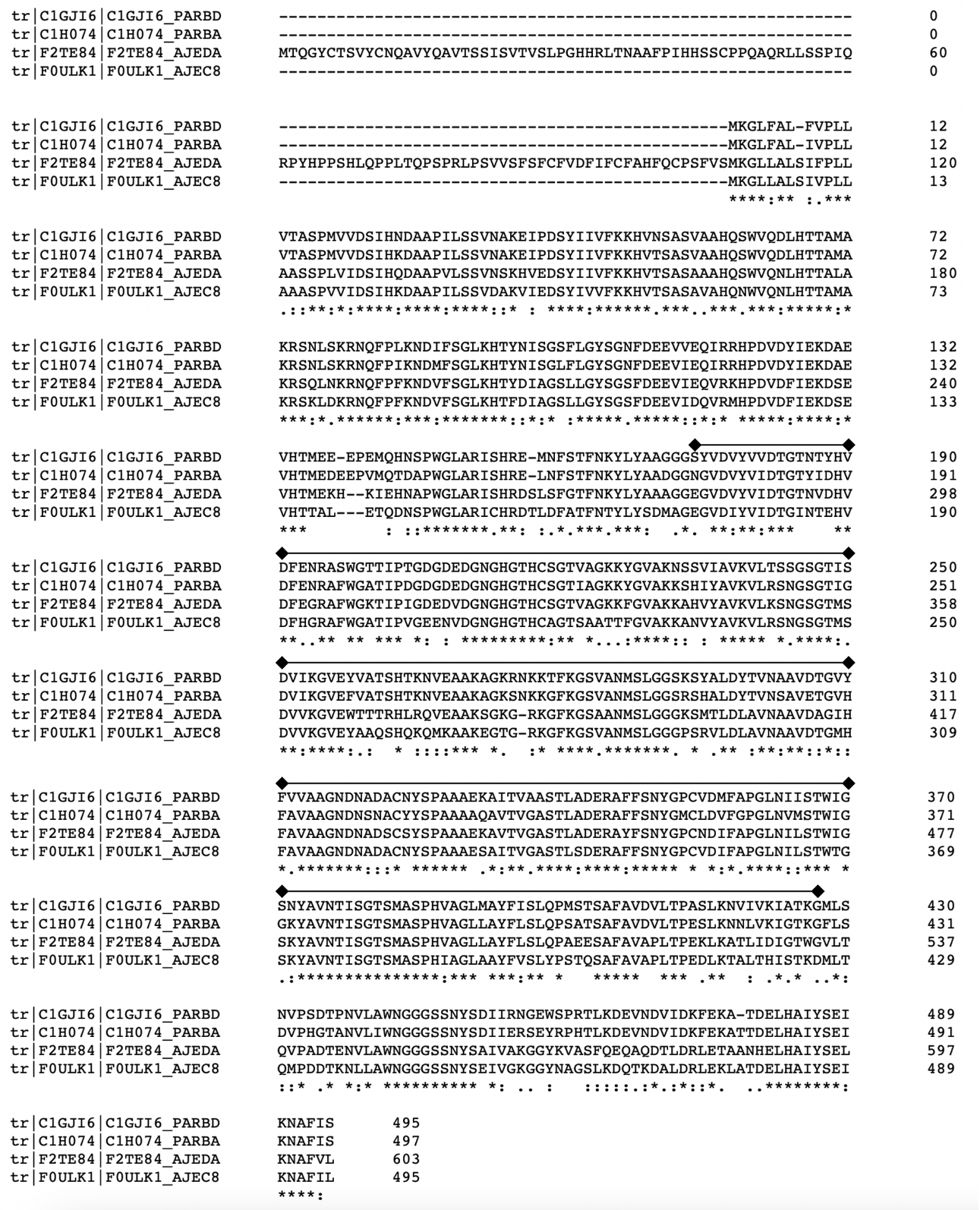


Figure S1: Multiple sequence alignment of PbSSP from PrP with other characterized secreted serine-proteases from dimorphic fungi. Similarity between serine-protease sequences from distinct dimorphic fungi was obtained by using the Clustal O (1.2.4) multiple sequence alignment tool. PbSSP (UniProt ID C1GJI6) identified in PrP was used as the reference sequence and it is shown at the first line. Sequences of serine-proteases from *Paracoccidioides lutzii* (UniProt ID C1H074), *Blastomyces dermatitidis* (UniProt ID F2TE84) and *Histoplasma capsulatum* (UniProt ID F0ULK1) are shown in lines 2, 3 and 4, respectively. *****, residues identical in all sequences tested. **:**, conserved substitutions. •, semi-conserved substitutions. ◆-◆ represents the subtilase domain for the reference sequence.
